# Neurocutaneous Melanosis in Association With Large Congenital Melanocytic Nevi in Children: A Report of 2 Cases With Clinical, Radiological, and Pathogenetic Evaluation

**DOI:** 10.3389/fneur.2019.00079

**Published:** 2019-02-07

**Authors:** Lei Chen, Liqin Zhai, Lika'a Fasih Y. Al-Kzayer, Shamil Naji Sarsam, Tingting Liu, Riyadh H. Alzakar, Yozo Nakazawa

**Affiliations:** ^1^Department of Pathology, Xinhua Hospital, Shanghai Jiaotong University School of Medicine, Shanghai, China; ^2^Department of Pathology, Shanxi Provincial People's Hospital, Taiyuan, China; ^3^Department of Pediatrics, Shinshu University School of Medicine, Matsumoto, Japan; ^4^Department of Radiology, Ibn Al-Nafees Hospital, Manama, Bahrain; ^5^Department of Pediatric Hematology/Oncology, Xinhua Hospital, Shanghai Jiaotong University School of Medicine, Shanghai, China; ^6^Department of Dermatology, Al-Hamdaniya General Hospital, Mosul, Iraq

**Keywords:** congenital melanocytic nevi (CMN), neurocutaneous melanosis (NCM), CNS melanoma, leptomeningeal melanoma, *NRAS* mutation, large-CMN, giant-CMN, *BRAF* V600E mutation

## Abstract

**Background:** Melanocytic nevi present at birth, or within the first few months of life, are defined as congenital melanocytic nevi (CMN). Neurocutaneous melanosis (NCM) is a rare disorder, represents pigment cell tumors of the leptomeninges, and occurs in association with large or multiple CMN. NCM carries an extremely poor prognosis. *NRAS* and *BRAF*^V600E^ genetic mutations were reported in CMN. Our aim was to report 2 rare cases of NCM associated with large-sized CMN.

**Materials and Methods:** Two cases were enrolled, a 19-month-old boy with multiple satellite and giant CMN (GCMN); and a 57-month-old girl with large CMN (LCMN). Both patients had central nervous system (CNS) symptoms, and therefore, were studied from clinical, radiological, and immunohistopathological aspects. Cytogenetic study was done for one of them.

**Results:** Both patients had CMN located in the head/neck, with no cutaneous melanoma. MRI was the most reliable method for early detection of NCM. NCM was proved in the 2 studied cases by immunohistopathology performed after surgery. The boy with GCMN carried *NRAS* mutation at codon 61, in addition to the characteristic facial features relevant to RASopathies. Both patients died despite surgical intervention.

**Conclusion:** Our report highlights the need for pediatricians to be alert to the risk of NCM in association with CMN, especially when a CMN lesion is large, or there are multiple satellite lesions, or the nevus location is at the head or neck. Moreover, in the setting of CMN, the absence of skin melanoma does not exclude the presence of NCM.

## Introduction

Melanocytic nevi present at birth, or within the first few months of life, are defined as congenital melanocytic nevi (CMN) (1). Most of CMN are relatively small measuring <1.5 centimeters (cm). Medium CMN are of 1.5 to <20 cm projected adult size (PAS), whereas large CMN (LCMN) are predicted to reach >20 cm PAS. Giant CMN (GCMN) are those >40 cm PAS (2).

Neurocutaneous melanosis (NCM) is a rare disorder characterized by the presence of benign or malignant pigment cell tumors of the leptomeninges in association with large or multiple CMN (3). Melanocytic cells are found in large number (in nodules or diffusely distributed) in the leptomeninges of the brain and/or spinal cord. High risk features of CMN that carry an increased risk of NCM include, large size, location at the posterior axis (head, neck, and paravertebral regions), and being multiple satellite lesions (1, 3–5).

Symptomatic NCM develops in 3–10% of infants and children with high-risk CMN and is associated with extremely poor prognosis (1, 6). Most patients with NCM presented in the first 2 years of life with evidence of central nervous system (CNS) manifestations of increased intracranial pressure, hydrocephalus, mass lesions, seizures, or spinal cord compression (7, 8). Asymptomatic NCM can be detected by screening magnetic resonance imaging (MRI) (6, 9, 10). Dandy-Walker malformation may be associated with NCM or occur in the absence of CNS involvement by melanocytic cells (1).

Genetic background showed that large-giant CMN harbor the somatic *NRAS* mutation at a frequency of around 95%. While *BRAF*^V600E^ somatic mutation was displayed mainly in small CMN, with a frequency of 88% (5, 11, 12). A previous report suggested that multiple CMN and NCM are caused by somatic mosaicism for *NRAS* mutation at codon 61 in a progenitor cell within the neuroectoderm of affected patients (12). The finding of the characteristic facial features in children with a CMN-positive *RAS* mutation similar to those of germline mutations in the RAS/RAF/MEK/ERK pathway, could link the “mosaic RASopathies” to the spectrum, as both germline and sporadic *RAS* mutations activate the samepathway (12–14).

Surgical intervention in the context of CNS-melanoma plays a role in relieving the symptoms and confirming the diagnosis, however, it is not curative (15).

Herein, we report 2 patients with large and giant CMN associated with symptomatic NCM. Clinical, radiological, and immunohistopathological features were evaluated in both cases, along with a cytogenetic study in one of them.

## Materials and Methods

Two cases were enrolled: a 19-month-old boy with multiple and GCMN treated in Xinhua Hospital at Shanghai; and a 57-month-old girl with LCMN from Shanxi Provincial People's Hospital. Both patients had CNS symptoms, and therefore, were studied thoroughly from clinical, radiological, and immunohistopathological aspects. Cytogenetic study was done for one case.

The immunohistopathological study was done using formalin-fixed, paraffin-embedded tissue samples, and staining with hematoxylin and eosin at initial diagnosis. Immunohistopathological evaluation of the CNS lesions was based on the 2016 World Health Organization (WHO) classification of CNS tumors. Immunohistochemical assays using a panel of monoclonal and polyclonal antibodies, including, HMB45 (clone HMB45, Long Island Biotec. Co., Shanghai, China), Melan-A (clone A103, Long Island Biotec. Co.), S100 (clone 4C4.9, Long Island Biotec. Co.), and Ki-67 (clone MIB-1, DAKO, Glostrup, Denmark), were performed. The immunohistochemical stain was considered positive when >25% of tumor cells were stained.

The cytogenetic evaluation was done for the boy using his affected brain tissue. Genomic DNA was extracted using a FFPE DNA kit (Amoy Diagnostics Co., Ltd., Xiamen, China). Tissue genotyping of *NRAS* in codon 61 and *BRAF*^V600E^ mutations, were conducted using ARMS Fluorescence Polymerase Chain Reaction (PCR) Diagnostic Kit (Amoy Diagnostics Co., Ltd., Xiamen, China) according to the manufacturer's instructions. FISH was performed on 3–4 μm thick sections of formalin-fixed paraffin-embedded tissue samples, employing P53 (LSI IGH/BCL2 dual-color dual-fusion probe, Vysis-Abbott, IL, USA). The cut-off values for the interphase FISH analyses were established, and for each sample, 100 evaluable nuclei with complete FISH signals were scored.

In accordance with the Declaration of Helsinki, written informed consent was obtained from the parents of each participants in this study for the information/data of the subjects to be used.

## Results

We calculated PAS for the boy (case-I) to be 42.9 cm, and for the girl (case-II) to be 20.4 cm, hence, the CMN size was defined as GCMN in case-I, and as LCMN in case-II. Both patients had no family history of CMN. NCM was proved in both patients by immunohistopathological evaluation performed after surgery, and thus, CNS melanoma (leptomeningeal melanosis and CNS melanosis) was confirmed, along with the exclusion of skin melanoma.

### Case-I

**Figure 1:**
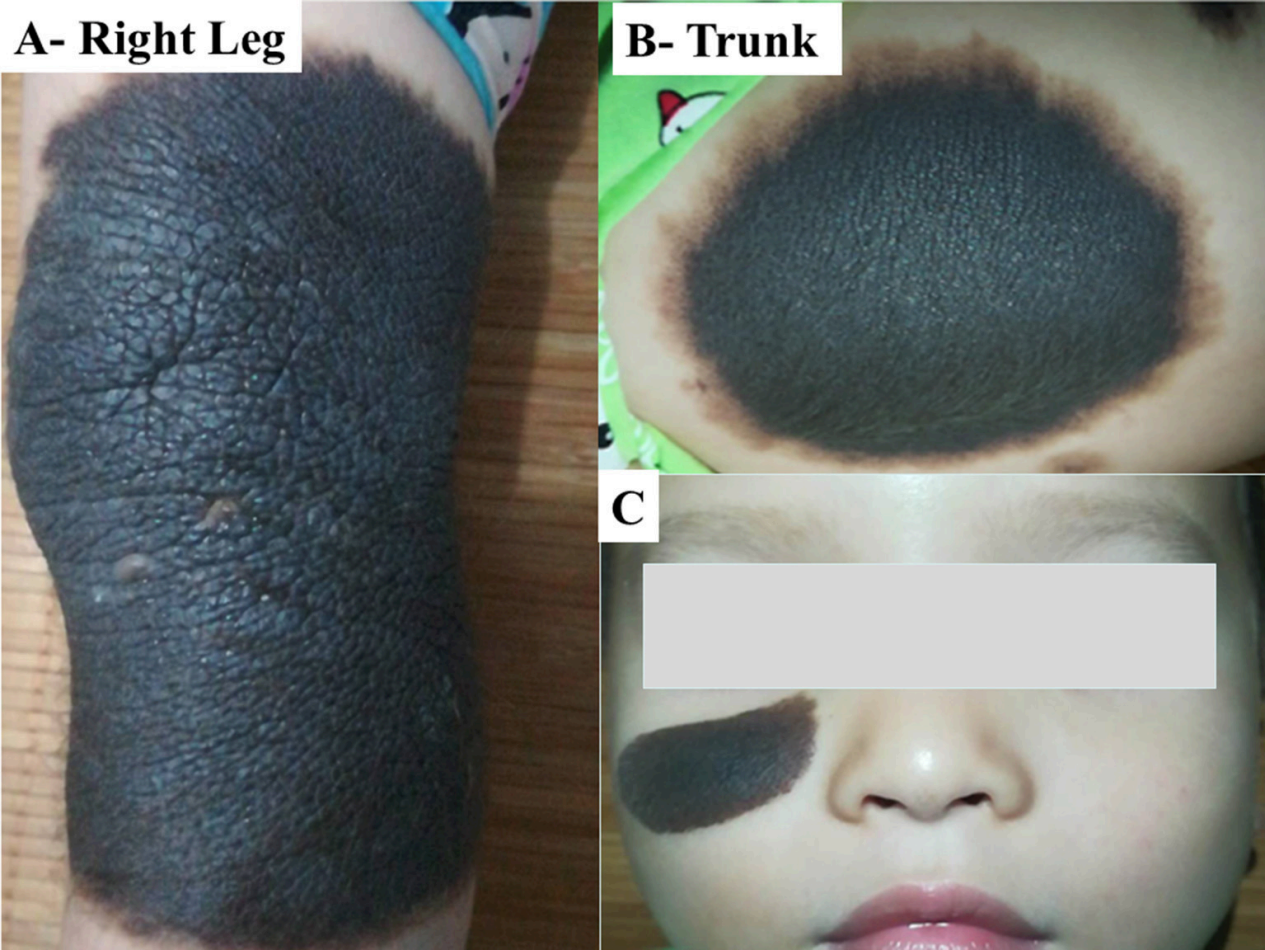
Photos of multiple and GCMN in case-I. CMN was found: **(A)** on the right leg of about 13 cm in diameter, **(B)** on the trunk, and **(C)** on the face. The boy had a round face, full cheeks, prominent forehead, hypertelorism, periorbital fullness, short nose, and everted lower lip.

**Figure 2:**
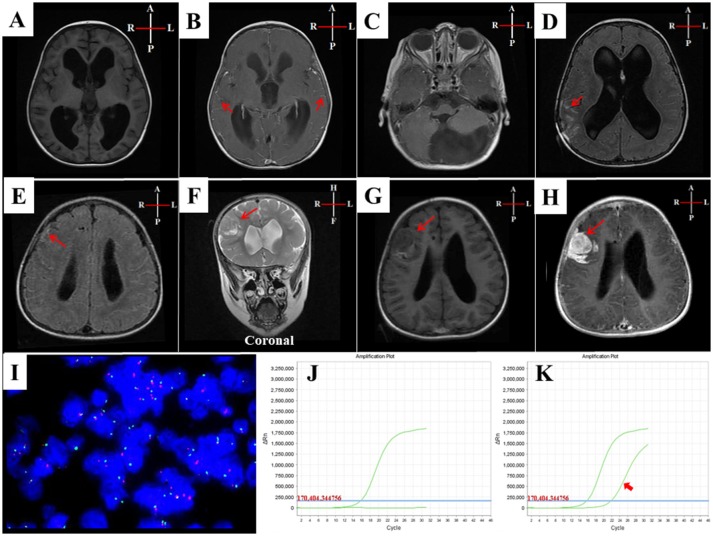
MRI and cytogenetic characteristics of case-I. **(A–H)** MRI of case-I: **(A)** T1W-axial-pre-contrast and **(B)** T1W-axial post-contrast, showed hydrocephalus of third and lateral ventricles with diffuse leptomeningeal enhancements (arrows in **B)**. **(C)** T1W-axial-post-contrast at posterior fossa level, showed a normal fourth ventricle with large arachnoid cyst at cisterna magna. **(D)** T1W-axial-post-contrast, 3 months and **(E)** 6 months later, showed evidence of ventriculoperitoneal shunt with persistence of the hydrocephalus and leptomeningeal enhancement and looked thick at the right parietal lobe in D and right frontal lobe in E (arrowed). **(F)** T2W-coronal image **(G)** T1W-axial image after 9 months, showed 3 cm extra-axial dural based intermediate signal lesion at the right frontal lobe surrounded by vasogenic edema causing mass effect on the right lateral ventricle and showed an intense enhancement in **(H)** T1W-axial-post-contrast image. **(I)** FISH analysis revealed a lack of allelic deletion of P53. **(J)** Genetic analysis of ARMS-PCR results detected no *BRAF*^V600E^ mutation. **(K)** *NRAS* mutation was shown in the third exon (codon 61) (arrowed).

A 19-month-old boy who had born in March 2015 with multiple brown-black skin pigmentation on the face, trunk, and right leg, with multiple satellite lesions (Figure 1). He was referred to hospital because of repeated vomiting for 4 days. He had a round face, full cheeks, prominent forehead, hypertelorism, periorbital fullness, short nose, and everted lower lip. The largest nevus was on the leg, of about 13 cm in diameter [(13 × 10), (8 × 6), (4 × 2) cm]. Enhanced MRI showed hydrocephalus (Figures 2A–H). A shunt surgery was done, to relieve the symptoms of intracranial hypertension, followed by MRI and/or computed tomography (CT) assessment every 3 months. The result of 6 months post-shunt MRI revealed the presence of supratentorial ventricular dilatation, brain stem volume reduction, in addition to leptomeningeal enhancement, however, no macroscopic mass was evident. Three months later, MRI showed a mass of 3 cm diameter, in the right frontal lobe. Although the tumor was completely resected, the patient died 4 months after surgery.

Pathological evaluation of CNS lesion, revealed that the mass was grossly dark-red to brown with the size of (4 × 4 × 2) cm. The tumor was firmly attached to the meninges. Microscopically, the tumor cells had atypical nuclei, obvious nucleoli, an increased karyoplasmic ratio, some mitoses, and remarkable necrosis, which infiltrated the brain parenchyma, accompanied with melanin deposition.

Immunohistopathological evaluation as shown in Supplementary Figure S1 disclosed that the tumor cells were positively expressing the antibodies of HMB45, Melan-A, and S100. Ki-67 was positively expressed in 30% of the cells, while P53 was negative.

Cytogenetic study using fluorescence *in situ* hybridization (FISH) revealed a lack of allelic deletion of P53. ARMS-PCR disclosed *NRAS* mutation in the third exon (codon 61), with a wild-type *BRAF*^V600E^ (Figures 2I–K).

### Case-II

A 4-year-and-9-months-old girl who had a brown-black skin pigmentation over her neck since birth, presented with headache and vomiting of 10 days duration. The largest diameter of the nevus was 12 cm (12 × 9.5 cm). Clinical examination revealed disturbed walking, balance, and coordination. CT and MRI disclosed a mass occupying most of the left cerebellar hemisphere, reaching the dura (Supplementary Figures S2A–D). She underwent complete surgical resection, however, she died 3 months after surgery, due to progressive disease.

Pathological evaluation of CNS lesion, revealed that the mass was grossly grayish-red to brown, of soft consistency, and the size of (4 × 3 × 2) cm. Microscopically, the tumor cells exhibited atypical nuclei, obvious nucleoli, and large number of mitoses, accompanied with outstanding necrosis and melanin deposition.

Immunohistopathological study showed that the tumor cells were positively expressing the antibodies of HMB45, Melan-A, and S100. Moreover, Ki-67 was positively expressed in 50% of cells (Supplementary Figures S2E–H).

## Discussion

NCM is rare and it encompasses both leptomeningeal melanosis and CNS melanosis (3). The prevalence of NCM in association with CMN is directly proportional to CMN size, number, and location. Nevi that arise in the posterior axis (head, neck, and trunk) have a higher risk of NCM (1, 16). Our 2 patients with NCM, had large-giant CMN, and head/neck location, with multiple satellite CMN in one case. Ruiz-Maldonado's proposed that the presence of satellites is a factor for upstaging GCMNs into a higher size category (17). Another study reported a new association between male gender and complications in CMN, independent of CMN-PAS and satellite lesions at birth (6). Thus, the boy in our report had all the predisposing factors for NCM.

The absolute risk of malignant melanoma is associated with the severity of the cutaneous phenotype, estimated as a 1–2% lifetime risk for all individuals with CMN, but rising to 10–15% in those with the LCMN (12).

A previous study showed that among 289 LCMN cases, 33 (11.4%) had manifested NCM all the 33 had their LCMN in a posterior axial location. In contrast, none of those with LCMN restricted to extremities had NCM. One possible explanation for the association of posterior axial LCMN and NCM is that if the melanocytic malformation occurs during the development and migration of the neural crest, from which both cutaneous melanocytes and leptomeningeal melanocytes originate, the risk that the malformation will involve both the CNS and the skin of the nearby posterior axis is increased. Malformations that result in nevi limited to the extremities presumably occur later, after migration of the melanocytes from the neural crest ventrally (4).

In agreement with the previous finding, case-I had mutated *NRAS* at codon 61, along with characteristic facial features relevant to RASopathies (5, 12, 13, 18, 19). The germline RASopathies have characteristic facial features, demonstrating the effect of RAS/RAF/MEK/ERK pathway imbalance on facial development (20). Given that NCM is generally caused by somatic *NRAS* mutation (18), and NCM is thought to represent an error in the morphogenesis of the embryonal neuroectoderm (7), such finding, of the characteristic facial features, has relevance as the neuroectoderm also contributes to the development of cartilage and bones of the face. Since both germline and sporadic *RAS* mutations activate the same pathway, it is not surprising to have similar phenotypic features (12). Importantly, in the case of NCM, the *NRAS* and *BRAF*^V600E^ genotyping, must be done using the affected brain tissue as well as the cutaneous lesion.

Similar to other reports, our cases with NCM had the symptoms related to increased intracranial pressure resulting from the obstruction of the ventricular system foramen, subarachnoid cistern, or arachnoid granulations secondary to melanocytic proliferations (1, 4, 7, 9).

There is no effective therapy in modifying the course of the disease, however, MEK inhibitors are under experimental evaluation (18). Surgery remains the primary option for symptomatic cases of NCM. Patients may be aided by palliative measures such as shunt placement to reduce intracranial pressure (7).

Clinical follow-up of CMN in children includes MRI screening when the CMN is defined as large-giant, being multiple satellites, and located in the posterior axis areas, even with no obvious CNS symptoms. Early imaging is optimal to provide the greatest sensitivity for diagnosis and to guide proper management (3). MRI is much more sensitive than CT for the detection of melanin, and it is the best method to evaluate the involvement of the leptomeninges (1). MRI displays melanotic brain lesions (T1 hyperintense and T2 hypointense compared with normal immature brain), resulting from the paramagnetic properties of stable free radicals within melanin pigment (3). However, MRI cannot distinguish benign from malignant melanocytes. Moreover, non-contrast MRI images lack sensitivity in detecting leptomeningeal melanocytic infiltration unless malignant degeneration has occurred; new presence of leptomeningeal enhancement or growth on sequential scans should raise suspicion for malignant transformation (3). On the other hand, MRI findings in asymptomatic NCM among children with GCMN are common and suggest an increased lifetime risk of CNS melanoma, though, they do not signify the eventual development of symptomatic NCM during childhood (21).

Furthermore, any child with a stepwise change in neurological/developmental symptoms or signs should have an MRI with the contrast of the brain and spine to look for new CNS melanoma (10).

The limitation of this report is the lack of genetic evaluation for the girl, as well as the photographic archiving.

## Conclusion

NCM, although rare, it is not uncommon in association with LCMN. Our report highlights the need for pediatricians to be alert to the risk of NCM in association with CMN, especially when a CMN lesion is large, or there are multiple satellite lesions, or the nevus location is at head or neck. Genetic evaluation in high-risk CMN is recommended. Moreover, in the setting of CMN, the absence of skin melanoma does not exclude the presence of NCM.

## Ethics Statement

The study was ethically approved by the Institutional Ethics committee of Xinhua Hospital affiliated to Shanghai Jiaotong University School of Medicine.

## Author Contributions

LC and TL conceptualized and designed the paper. LC and LZ performed the immunopathological evaluation and related experiments and wrote the paper. LA-K evaluated the clinical data, and references. TL, LA-K, and SS prepared the figures and wrote the paper. RA and YN evaluated and revised the paper. All authors read and approved the final manuscript.

### Conflict of Interest Statement

The authors declare that the research was conducted in the absence of any commercial or financial relationships that could be construed as a potential conflict of interest.

## References

[1] Sawicka E Szczygielski O Zak K Peczkowski P Michalak E Bekiesinska-Figatowska M. Giant congenital melanocytic nevi: selected aspects of diagnostics and treatment. Med Sci Monit. (2015) 21:123–32. 10.12659/MSM.891279 25577155 PMC4298998

[2] Krengel S Scope A Dusza SW Vonthein R Marghoob AA. New recommendations for the categorization of cutaneous features of congenital melanocytic nevi. J Am Acad Dermatol. (2013) 68:441–51. 10.1016/j.jaad.2012.05.043 22982004

[3] Jakchairoongruang K Khakoo Y Beckwith M Barkovich AJ. New insights into neurocutaneous melanosis. Pediatr Radiol. (2018) 48:1786–96. 10.1007/s00247-018-4205-x 30074086 PMC7469866

[4] DeDavid M Orlow SJ Provost N Marghoob AA Rao BK Wasti Q. Neurocutaneous melanosis: clinical features of large congenital melanocytic nevi in patients with manifest central nervous system melanosis. J Am Acad Dermatol. (1996) 35:529–38. 8859278 10.1016/s0190-9622(96)90674-x

[5] Charbel C Fontaine RH Malouf GG Picard A Kadlub N El-Murr N. NRAS mutation is the sole recurrent somatic mutation in large congenital melanocytic nevi. J Invest Dermatol. (2014) 134:1067–74. 10.1038/jid.2013.429 24129063

[6] Kinsler VA Chong WK Aylett SE Atherton DJ. Complications of congenital melanocytic naevi in children: analysis of 16 years' experience and clinical practice. Br J Dermatol. (2008) 159:907–14. 10.1111/j.1365-2133.2008.08775.x 18671780

[7] Kadonaga JN Frieden IJ. Neurocutaneous melanosis: definition and review of the literature. J Am Acad Dermatol. (1991) 24:747–55. 1869648 10.1016/0190-9622(91)70115-i

[8] Islam MP. Neurocutaneous melanosis. Handb Clin Neurol. (2015) 132:111–7. 10.1016/B978-0-444-62702-5.00007-X 26564074

[9] Kinsler VA O'Hare P Bulstrode N Calonje JE Chong WK Hargrave D. Melanoma in congenital melanocytic naevi. Br J Dermatol. (2017) 176:1131–43. 10.1111/bjd.15301 28078671 PMC5484991

[10] Waelchli R Aylett SE Atherton D Thompson DJ Chong WK Kinsler VA. Classification of neurological abnormalities in children with congenital melanocytic naevus syndrome identifies magnetic resonance imaging as the best predictor of clinical outcome. Br J Dermatol. (2015) 173:739–50. 10.1111/bjd.13898 25966033 PMC4737261

[11] Ichii-Nakato N Takata M Takayanagi S Takashima S Lin J Murata H. High frequency of BRAFV600E mutation in acquired nevi and small congenital nevi, but low frequency of mutation in medium-sized congenital nevi. J Invest Dermatol. (2006) 126:2111–8. 10.1038/sj.jid.5700366 16691193

[12] Kinsler VA Thomas AC Ishida M Bulstrode NW Loughlin S Hing S. Multiple congenital melanocytic nevi and neurocutaneous melanosis are caused by postzygotic mutations in codon 61 of NRAS. J Invest Dermatol. (2013) 133:2229–36. 10.1038/jid.2013.70 23392294 PMC3678977

[13] Bekiesinska-Figatowska M Sawicka E Zak K Szczygielski O. Age related changes in brain MR appearance in the course of neurocutaneous melanosis. Eur J Radiol. (2016) 85:1427–31. 10.1016/j.ejrad.2016.05.014 27423683

[14] Hafner C Groesser L. Mosaic RASopathies. Cell Cycle (2013) 12:43–50. 10.4161/cc.23108 23255105 PMC3570515

[15] Subbiah V Wolff JE. Rapid response to therapy of neurocutaneous melanosis with leptomeningeal melanoma. Pediatr Blood Cancer (2010) 54:180–1. 10.1002/pbc.22279 19722276

[16] Krengel S Hauschild A Schäfer T. Melanoma risk in congenital melanocytic naevi: a systematic review. Br J Dermatol. (2006) 155:1–8. 10.1111/j.1365-2133.2006.07218.x 16792745

[17] Ruiz-Maldonado R. Measuring congenital melanocytic nevi. Pediatr Dermatol. (2004) 21:178–9. 10.1111/j.0736-8046.2004.21222.x 15078366

[18] Pawlikowski JS Brock C Chen SC Al-Olabi L Nixon C McGregor F Acute inhibition of MEK suppresses congenital melanocytic nevus syndrome in a murine model driven by activated NRAS and Wnt signaling. J Invest Dermatol. (2015) 135:2902 10.1038/jid.2015.230 26178707

[19] Halaban R Krauthammer M. RASopathy gene mutations in melanoma. J Invest Dermatol. (2016) 136:1755–59. 10.1016/j.jid.2016.05.095 27236105 PMC4992636

[20] Zenker M. Clinical manifestations of mutations in RAS and related intracellular signal transduction factors. Curr Opin Pediatr. (2011) 23:443–51. 10.1097/MOP.0b013e32834881dd 21750428

[21] Foster RD Williams ML Barkovich AJ Hoffman WY Mathes SJ Frieden IJ. Giant congenital melanocytic nevi: the significance of neurocutaneous melanosis in neurologically asymptomatic children. Plast Reconstr Surg. (2001) 107:933–41. 10.1097/00006534-200104010-00005 11252085

