# Supplementary figures and images for: Neurocutaneous Melanosis in Association With Large Congenital Melanocytic Nevi in Children: A Report of 2 Cases With Clinical, Radiological, and Pathogenetic Evaluation
Source: Front Neurol. 2019 Feb 7;10:79. doi: 10.3389/fneur.2019.00079 (PMC6374324; doi:10.3389/fneur.2019.00079)

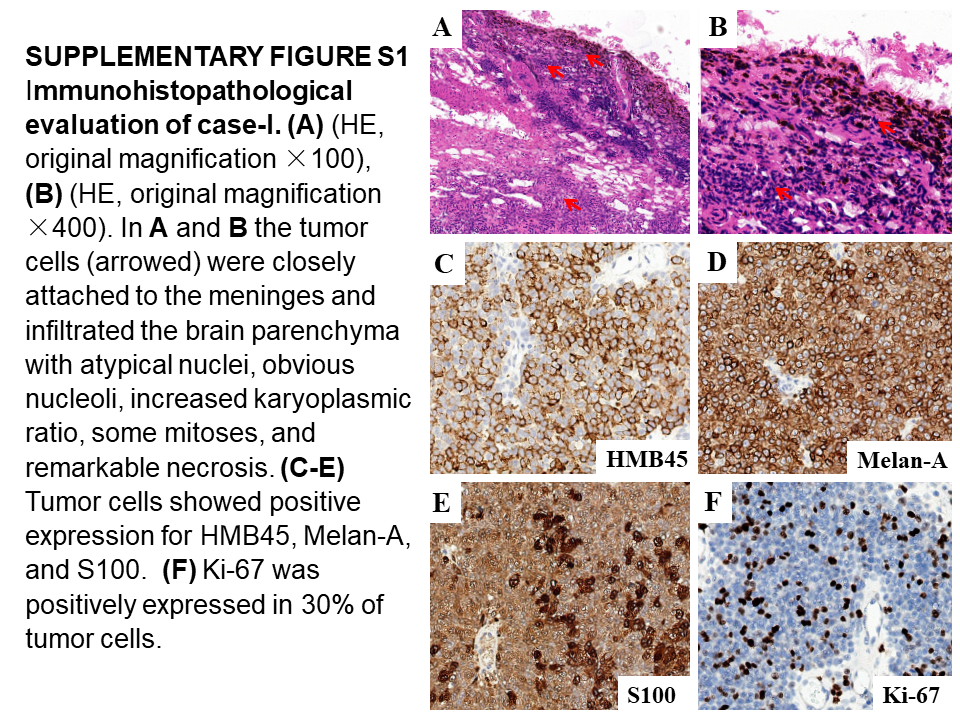

Supplement: Supplementary file 1 [file Image_1.tif]

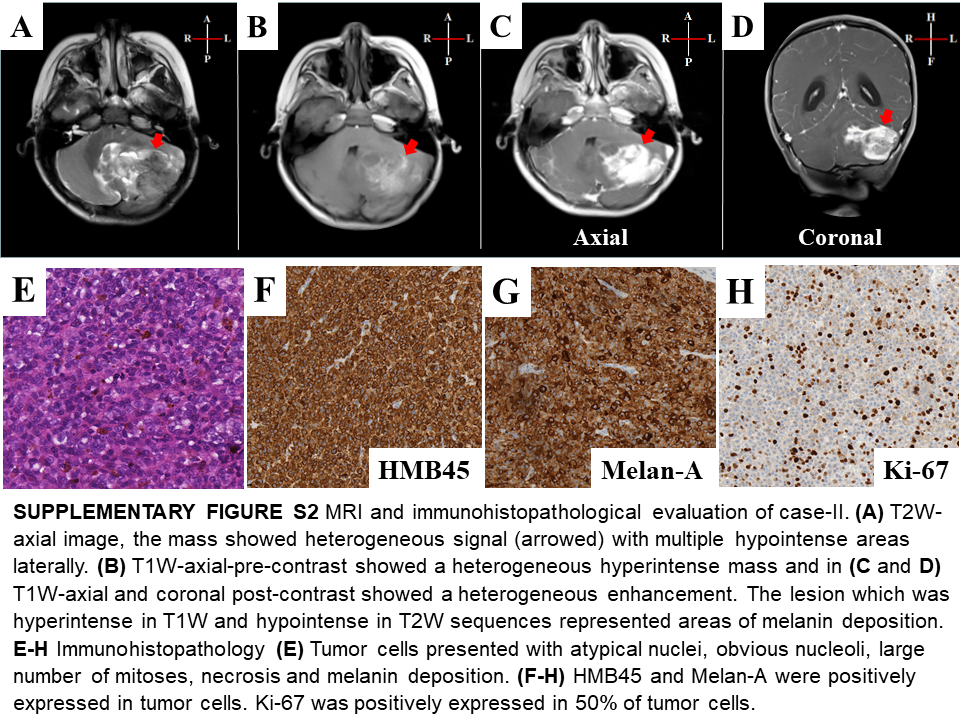

Supplement: Supplementary file 2 [file Image_2.tif]
